# Potential Risk of Pollen from Genetically Modified MON 810 Maize Containing Cry1Ab Toxin to Protected Lepidopteran Larvae in the Pannonian Biogeographical Region—A Retrospective View

**DOI:** 10.3390/insects13020206

**Published:** 2022-02-17

**Authors:** Béla Darvas, Gergő Gyurcsó, Eszter Takács, András Székács

**Affiliations:** 1 Hungarian Society of Ecotoxicology, H-1022 Budapest, Hungary; 2 Agro-Environmental Research Centre, Institute of Environmental Sciences, Hungarian University of Agriculture and Life Sciences, H-1022 Budapest, Hungary; gyurcso.gergo@uni-mate.hu (G.G.); takacs.eszter84@uni-mate.hu (E.T.); szekacs.andras@uni-mate.hu (A.S.)

**Keywords:** MON 810, Cry1Ab toxin, protected caterpillars, *Urtica dioica*, *Nymphalis io*, *Nymphalis c-album*, *Vanessa atalanta*, maize pollen distribution, Cry1Ab toxin resistance

## Abstract

**Simple Summary:**

The Cry1Ab toxin content in different plant organs is highly variable by genetic events and cultivars. This applies particularly to the pollen, which is the main route of exposure of protected lepidopteran larvae. Thus, uncertainties appear regarding safety assessments on the basis of analytical and biological studies: (*a*) genetic events and cultivars produce various Cry1Ab toxin contents; thus, risk analyses based on single pollen counts may be erroneous; (*b*) analytical problems have been identified explaining the high variability of the documented pollen toxin content; (*c*) stinging nettle patches subject to maize pollen sedimentation are frequent nearby maize field edges, where protected nymphalid larvae may feed; (*d*) substantial maize pollen sedimentation (300–600 pollen grains/cm^2^ in the dominant wind direction) occurs on the leaves of stinging nettle; (*e*) July and August are the critical months for the young larvae of *Nymphalis io*, which are the most sensitive for pollen containing Cry1Ab toxin; (*f*) the exposure of these larvae to maize pollen containing >100 ng of Cry1Ab toxin/g results in <40% mortality and extended developmental times in younger stages. This is a definite hazard, which is a sufficient legal ground for habitat conservation of this protected species in Hungary.

**Abstract:**

A credible risk analysis of maize pollen containing Cry1Ab toxin must include the assessment of (*i*) pollen production and its Cry1 toxin content; (*ii*) distribution of the pollen grains in the surroundings; (*iii*) pollen-catching capacity of the weeds on field edges; (*iv*) the lifestyle of protected lepidopteran larvae living on weeds; (*v*) Cry1 toxin sensitivity of non-target caterpillars; and (*vi*) Cry1 toxin resistance of individual non-target populations. The concentration range of 5–4300 ng Cry1Ab toxin/g dry pollen determined in MON 810 pollen batches is too diverse for handling it as a single set in any mathematical modeling. Within the work carried out mainly with the DK-440 BTY cultivar, the seed samples officially received from the variety owner produced significantly different (250–470 vs. 5–15 ng/g) Cry1Ab toxin concentrations in the pollen. *Nymphalis io* L1-L3 larvae were nearly six times more sensitive for Dipel than *Nymphalis c-album*. Feeding on the back side and in a leaf nest, *Vanessa atalanta* may be subject to lower pollen exposures. *N. io* larvae may actively attempt to avoid patches with high pollen contamination. Cry1Ab toxin resistance also partially emerged in *N. io* populations reared in the Pannonian Biogeographical Region (Hungary).

## 1. Introduction

Pollen containing Cry1Ab toxin ingested by non-target lepidopteran larvae that consume weeds emerging in maize fields has received wide attention by conservation biologists. Danaus plexippus larvae, reared on Asclepias syriaca leaves dusted with the pollen of “Bt maize” (N4640 maize, SYN-BT11-1—Cry1Ab), reduced their feeding activity, showed delayed growth, and suffered higher mortality than larvae reared on leaves dusted with maize pollen without Cry1Ab toxin [1]. Several commentaries [2,3,4,5] followed this pioneering paper, emphasizing that the toxicity observed is a more complex phenomenon than thought earlier, due to the interplay among various factors, including natural maize pollen distribution, Cry/Vip toxin content of different genetic events of maize (presently: ACS-ZM4-3—Cry9C, DAS-01507-1—Cry1Fa, DKB-89614-9—Cry1Ac, MON-810-6—Cry1Ab, MON-89034-3—Cry2Ab2 + Cry1A.105; SYN-BT11-1—Cry1Ab, SYN-IR162-4—Vip3Aa20, etc.) and cultivars, species sensitivity to Cry/Vip toxin, and the conservation status of lepidopteran species living in Europe.

After Hungary joined the European Union (EU), the EU natural heritage became enriched by a unique region constituting a biogeographical unit, namely, the Pannonian Biogeographical Region. In the Pannonian Biogeographical Region, representing 3% of the total area of the EU, there are 55 habitat types of Community interest, representing as much as 26% of the protected habitats in the EU [6]. Most (85%) of the Pannonian Biogeographic Region is located in Hungary. In Hungary, 179 Lepidoptera species are protected by national law [5], an additional 19 species are rigorously protected, and 16 other species of Community interest are under strict regulation [7,8], which is unique even in European practices of conservation biology. Nevertheless, Hungary is a leading maize producer after France in the EU, holding pronounced interests in the very sensitive maize seed production, which has led to exceptionally cautious views regarding the cultivation of genetically modified (GM) plants [9].

MON 810—producing Cry1Ab toxin [10,11,12,13,14,15,16]—authorized for cultivation in the EU is resistant to *Ostrinia nubilalis* and *Helicoverpa armigera*. There has been an ongoing formal debate on non-target environmental effects, resulting in several national moratoria. Economic reasons, such as the technology fee for patents, the contract system restricting farmers’ rights, concerns regarding the narrowing of national variety choices, practical problems of field treatment and consumer aversion all play roles in rendering MON 810 the only genetic event in the EU approved for public cultivation. Only Spain and Portugal have authorized and cultivated MON 810 maize cultivars in practically important field sizes. It reached ~30% of maize production in Spain during 2010–2015. Our aim with this retrospective view based on our earlier published studies [5,9,10,11,12,13,17,18], referred to by some controversial European model ([19] vs. [20,21,22]) on risk analysis and used by European Food Safety Authority for its statements, is to reassess this issue and recalculate our results due to the emotionally based political battles in the EU.

**Figure 1:**
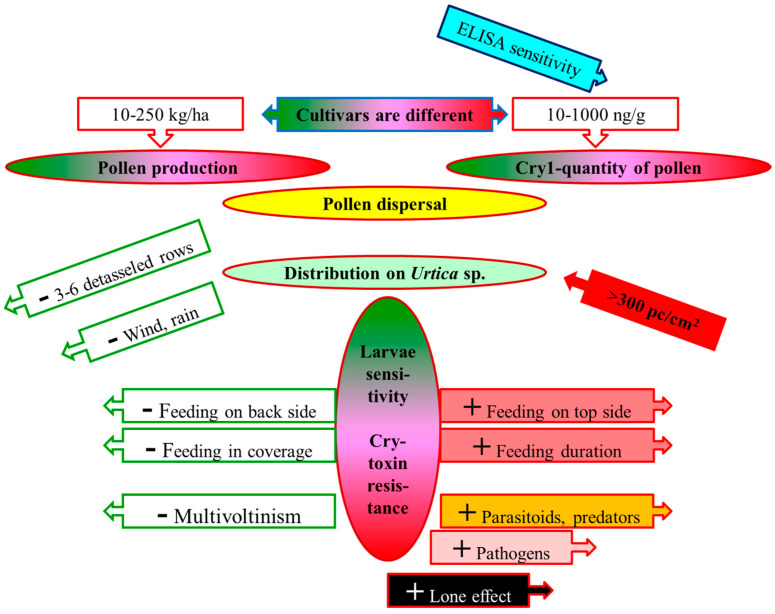
Main elements of a risk analysis regarding lepidopteran larvae feeding on *Urtica* species contaminated with sedimented MON 810 pollen (sketch) Notes: **+** increases the risk; **–** decreases the risk.

A credible risk analysis regarding maize pollen (depicted in Figure 1) must include the assessment of: (i) pollen production and Cry/Vip toxin content in various cultivars; (ii) distributions of the pollen grains in the surroundings; (iii) pollen-catching capacity of the leaves of weeds on field edges; (iv) the lifestyle of rare/protected lepidopteran larvae living on critical weeds; (v) Cry/Vip toxin sensitivity of non-target lepidopteran larvae; and (vi) Cry toxin resistance of individual non-target subpopulations.

## 2. Pollen Production and Cry1Ab Toxin Content of MON 810 Cultivars

Average anther numbers vary between 2400 and 4100 per plant in maize cultivars, showing a great variability among cultivation sites and years. In turn, the potential pollen yield was calculated to be 160–220 kg dry pollen/ha, considering an average pollen weight of 2.5 × 10^−7^ g/dry pollen grain (4000 dry pollen grains weighing 1 mg) and a pollen amount of approximately 3000 pollen grains/anther. In the very dry year of 2002, when only the lead branch of the tassel flowered, pollen production of DK-440 BTY maize was nearly 40 kg dry weight/ha near Budapest (Nagykovácsi) [17]. Pollen production of maize cultivars can be widely variable among varieties in which the male parent is event MON 810 and the female partner is a well-adapted national cultivar (e.g., Pactol vs. Pactol CB, DK-440 vs. DK-440 BTY, Nobilis vs. Novelis). In addition, weather conditions, particularly the quantity of rain prior to tassel formation, also strongly affect pollen production [17,23].

**Figure 2:**
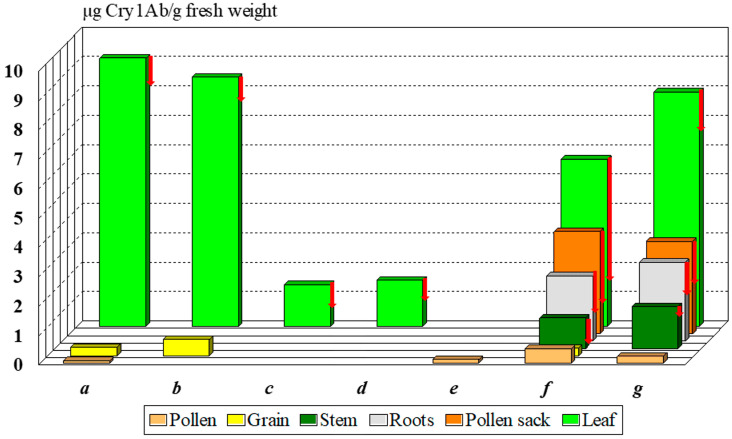
Average Cry1Ab toxin content in various organs of MON 810 maize cultivars reported by different authors. Notes: red arrows show the minimum values; a—[24], b—[25], c—[26], d—[27], e—[29], f—[28], g—[11].

The Monsanto documentation, registered in the databases of AGBIOS, specifies the Cry1Ab toxin content of MON 810 8–11, 0.1, and 0.2–0.9 µg Cry1Ab/g fresh weight in the leaf, the pollen, and the seeds [24,25]. Other authors reported substantially different [26,27] or similar values [11,28] (Figure 2). The Cry1Ab toxin content of pollen is considered low relative to the leaves, but its variability [24,28,29] is high: 0.01–0.49 µg Cry1Ab/g fresh pollen. Lang et al. [29] and Nguyen and Jehle [28] measured dry pollen samples. What is the reason for the high variability?

### 2.1. Differences between Genetically Modified Cultivars

The non-GM (called near-isogenic) female line may cause significant differences between compositions of cultivars. Data depicted on Figure 2 illustrate this characteristic difference among cultivars, as the near isogenic line was different in every case indicated. Bruns and Abel [26] and Abel and Adamczyk [27] tested a Pioneer (DuPont → Corteva) hybrid, Nguyen and Jehle [28] worked with a Novelis (Monsanto → Bayer) cultivar, and Székács et al. [11] experimented with a DeKalb (Monsanto → Bayer) cultivar. The last classical steps (i.e., ♀Nobilis + ♂MON 810 → Novelis; ♀DK-440 + ♂MON 810 → DK-440 BTYੑ) in seed production may change several parameters of Cry1Ab toxin production. In our case, in 2001 and 2004, we obtained two DK-440 BTY seed samples, both originating from Monsanto Hungary. Significant differences were found in Cry1Ab toxin production in the pollen of these two varieties of a single maize cultivar of MON 810 in two lots of the same cultivar (termed DK-440 BTY^A^ and DK-440 BTY^B^ in this paper for distinction) to be 470 and 5-20 ng Cry1Ab toxin/g dry pollen, respectively [11,17,18]. Thus, the first seed lot (DK-440 BTY^A^) of the two allotments received from the variety owner, both labeled “DK-440 BTY”, produced significantly more Cry1Ab toxin in the leaves, roots, and pollen sack as well [11,12,30] than the second shipment (DK-440 BTY^B^). The great variety in Cry1Ab toxin content indicates that the pollen density alone is not sufficient to clearly characterize the toxicological effects of MON 810 maize pollen on sensitive lepidopteran larvae [19,20,21]. Toxin content in the pollen must be also taken into consideration. In contrast, most authors in the scientific literature handled risk assessments solely based on pollen density. This has been the most considerable, although not the only, problem with early risk analysis [19,20,21]. We have described the levels of and the variability in Cry1Ab toxin content in MON 810 maize [11,12,16,30,31], but variations in the levels of this toxin can be even greater in other genetic events. SYN-EV176-9 maize pollen contains nearly ten to fifty times more Cry1Ab toxin/g pollen than MON 810, on average. The term “Bt maize” (GM maize expressing *Bacillus thuringiensis* endotoxin(s), e.g., Cry1Ab) is rather broad both from analytical and toxicological aspects: the Cry1Ab toxin content in different organs (including pollen) is highly variable by genetic event and cultivar. The concentration range of 5–4300 ng Cry1Ab toxin/g pollen ([12,16,17,18,24,25] vs. [19,20,21,22]) determined in MON 810 pollen batches is too diverse for handling it as a single set in any mathematical modeling. Not considering the high differences in the toxin content in pollen, and thus ignoring the data originating from corresponding analytical studies, is the key problem with the present risk analysis of non-target Lepidoptera to Bt maize pollen [19,20,21,22].

### 2.2. Cry1Ab Toxin Production Changes during Plant Development

The toxin concentration was found to show a rapid rise in the leaves by the end of the 5th week of cultivation, followed by a gradual decline by the 16th week and a slight increase again during the last 2 weeks due to partial desiccation. Similar, but smaller fluctuations of toxin levels were seen in the roots during plant development. In contrast, Cry1Ab toxin levels appeared to be stable in the stem, anther wall, pollen, and grain (Figure 2) [11,12,13,16,30,31].

Cry1Ab toxin content was significantly reduced in leaves at the lowest leaf level, compared with the higher leaf levels, due to partial leaf necrotization. A substantial (up to 22%) plant-to-plant variation in Cry1Ab contents in the leaves was observed. When studying toxin distribution within the cross and longitudinal sections of single leaves, less variability was detected diagonally, with an approximately 20% higher toxin concentration at or near the leaf vein. More significant variability was seen lengthwise along the leaf at the sheath and rising to a maximum concentration at the middle of the lamella. Cry1Ab toxin contents may exhibit significant decreases toward the leaf tip due to necrotization [12].

### 2.3. Analytical Difficulties of Cry1Ab Determination

Widely used analytical methods for the detection of Cry toxins are enzyme-linked immunosorbent assay (ELISA—Envirologix, Abraxis, etc.) systems. Reported Cry1Ab toxin concentrations in MON 810 maize show high variability: order of magnitude differences have been observed among various plant parts from different varieties, those cultivated at different locations, and sometimes even within the same plant variety at a single location. In addition to being biological sources of variability, numerous analytical problems have been identified explaining the high variability among the documented data on toxin content. Two fundamental difficulties of analytical determinations of Cry1Ab toxin in Bt plants have been highlighted: the problem of the quantitative detection of plant-produced preactivated toxin (a 91 kDa molecular weight N-terminal fragment of the protoxin) with ELISAs based on protoxin-specific (a 131 kDa molecular weight microbial toxin) antibodies, on the one hand, and the calibration difficulty of commercial ELISA systems with linear regression instead of the sigmoid calibration typical for immunoassays, on the other hand. In addition, results obtained with different ELISA methods are often not directly comparable with each other [13,16,30,31].

## 3. Frequent Weeds on Maize Field Edges in the Pannonian Biogeographical Region and Their Maize Pollen-Catching Capacity

The stinging nettle (*Urtica dioica*) is a common plant species living at areas adjacent to maize fields in Hungary, and certain sensitive lepidopteran larvae develop on this weed [5,32]. These protected caterpillars can sporadically live on Rubus spp. [17] and thorn apple (Datura stramonium) as well; however, these latter assemblages are rarely found in Hungary.

In our experiments, carried out at Zsámbék, Hungary, we determined pollen densities of 190, 328, 339 and 1114 pollen grains/cm^2^ at the same time and leaf level in maize, stinging nettle, thorn apple and marshmallow (Althea officinalis), respectively [17]. The pollen-catching capacity of plant leaves is maximal if the size of the sticky hairs is nearly the same as the pollen grains (diameter 70–100 µm), such as in Urtica species. Thus, the pollen-retaining features of stinging nettles are better than those of maize, but worse than marshmallow. After 1 month (wind and rain effects), marshmallow retained nearly half of the maize pollen caught on the leaf surface, the corresponding pollen-retaining capacity of stinging nettle was 13%, whereas that of maize and thorn apple were as low as 0–1% [17].

The leaf surface/biomass ratio of U. dioica (the host plant of *Nymphalis io* larvae) is 2.8-times higher than that of Senecio jacobae (the host plant of Danaus plexippus larvae). *N. io* larvae ingest 7 or 35 pollen grains along with consumed host leaf material during the first larval instars in cases of 100 or 500 pollen cm^2^ maize pollen densities on stinging nettles, respectively [17]. Larvae in the last instars of *N. io* consumed a leaf biomass corresponding to 2600-times more surface with maize pollen grains than the 1st instar [recalculated from 17], while the corresponding value for *V. atalanta* is 2100.

## 4. Maize Pollen Distribution

Maize pollen is released during periods of dry air weather conditions. Maize pollen is typically shed from a single plant within 8 days, with a difference of 8 days between pollen shed in a maize population from the first and last individuals, corresponding to an average of approximately 16 days of the male flowering period within a single maize field [23,32,33,34,35,36]. The duration of pollen shedding may be doubled, or sometimes even tripled, especially at field edges with weedy perimeters, where herbicide treatments are not very effective.

### 4.1. Pollination Time of Maize in the Pannonian Biogeographical Region

According to a decade-long experience [5,18,31,32], individual maize plants are pollinated in 4–8-day periods annually. At the field level, pollination lasts 7–21 days. The pollination variability of the individual plants was particularly strong in the first 5 m of the field (i.e., edge effect). Maize hybrids had shorter pollination periods (7–14 days) than the open pollinated local variety. The whole maize pollination period—for different varieties with the FAO number assortment in a country—is rather wide. The pollination time depends on the time of sowing, the soil temperature during seed emergence, soil quality, water management and weather conditions. Maize pollination is frequent during the second half of July and August in the Pannonian Biogeographical Region. The duration of maize pollination is important for caterpillar larval stages living on *Urtica* spp. This defines the time window for this exposure.

### 4.2. Average Pollen Density of Maize Cultivars

Pollen densities detected on stinging nettle were in the same range (frequently found to be 230–350 pollen/cm^2^) that was determined on maize leaves at similar leaf levels. We have never detected such a high pollen density [5,17,18,32] as that which had been reported by Fahse et al. in their mathematical model [21]. The *N. io* larvae actively avoid feeding on leaf patches with pollen densities over 1000 pollen grains/cm^2^ (practically very rare cases, mostly near the main leaf vein), and for this reason, dose-dependence does not exist in the case of maize pollen with Cry1Ab toxin content. In the case of Dipel (a bioinsecticide with Cry1 toxin as active ingredient), the situation is very different [17]. The protein content of MON 810 maize pollen (not identical to that of Dipel) activates the feeding sensors of the larvae, which triggers them to avoid the leaf surfaces covered heavily with maize pollen. Thus, maize pollen alters the host plant quality for *N. io* larvae. Not considering this effect is also an important weakness all mathematical models presented in this field [19,20,21,22].

## 5. Protected Lepidopteran Larvae near Edge of the Maize Field

Exposure to pollen containing the transgenic Cry1Ab toxin may exert detrimental effects on non-target lepidopteran species as well. Direct mortality as an acute toxicity sign of Cry1 toxin is well known. Chronic mortality types such as longer developmental times, and sometimes lower pupal weights, may also occur [5,17,18]. The likelihood of predation (mostly by insectivorous birds), parasitoids and pathogens becomes elevated when the larval development is slow. We frequently observed that *N. io* larval populations are regularly reduced by a viral pathogen (cypovirus 2) and certain parasitoids (*Sturmia bella*, Tachinidae and *Pteromalus puparum*, Pteromalidae) in Hungary [17,18,37]. During a 12-year experimental period, all the MON 810 pollen-treated larvae were infected by cypovirus 2 on one occasion, and *N. io* pupae were ~60% infected by *P. puparum* in different years.

### 5.1. Lifestyles of Protected Lepidopteran Larvae Living on Urtica Species

The peacock butterfly (*N. io*), comma butterfly (*Nymphalis c-album*), small tortoiseshell (*Aglais urticae*) and red admiral (*V. atalanta*) are protected species in Hungary. The map butterfly (*Araschnia levana*) is another nymphalid larva that also feeds on *Urtica* species, but it does not have a protected status in Hungary. In cases of protected species (this legal status may change nation by nation), no risk is acceptable (can be tolerated), and in these cases, the host plant quality needs to be undisturbed. Maize pollen containing Cry1 toxin cannot comply with the protected status, because it changes the host plant quality of *Urtica* species at maize field edges and may cause disturbances in the larval development. Thus, MON 810 maize cannot constitute a part of integrated pest management [38]. Living on nettles, *Aglais urticae* is also a controversial species which showed only negligible effects for *Bt* maize pollen (without Cry1 toxin measurement) [39,40].

**Figure 3:**
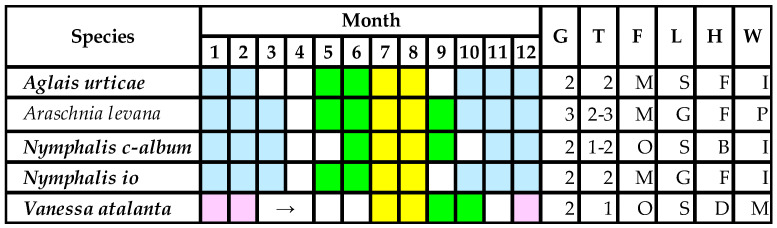
Larval stadia of nymphalid species developing on *Urtica* spp. in the Pannonian Biogeographical Region [5,17,18,32]. Notes: ***Italics bold***—protected species; colors: yellow—maize pollination, green—untreated population, white—active imago, blue—larval diapause or hibernation of imago, purple—migration to South Europe; letters: G—generation number, T—maize pollen-treated generation number, F—feeding type (M—monophage, O—oligophage, P—polyphage), L—larval life style (S—solitary larva, G—larvae in group), H—feeding habits (B—back part of the leaf, F—front part of the leaf, D—larva shelters in leaf web), W—overwintering (P—pupa, I—imago, M—no overwintering strategy, imago migration).

In cases of multivoltine species—*A. urticae*, *N. io*, *N. c-album*, *V. atalanta*, which has two generations in Hungary—only half of the populations are exposed to maize pollen [5,17,32]. Only monophagous species have no chance to develop on other host plants (Figure 3). Nevertheless, not all *Urtica* populations are settled down the edge of maize fields. Therefore, only a small part of the larval populations is at a possible hazard. This is another fact which cannot be significantly assessed by the present risk analysis [19,20,21,22].

Caterpillar lifestyles (Figure 3) may heavily alter the effects of DK-440 BTY^A^ pollen. In the case of young *V. atalanta* larvae (L1–L3), leaves have been rolled and, in this shelter, larvae do not reach the maize pollen settled. Referring to our study [5], Perry et al. [19] considered *V. atalanta* and *N. io* as having equal larval sensitivity to pollen containing Cry1Ab toxin; however, our referred article did not mention the name of this species at all. It has also been strange that they refereed to us when stating that the first instar is the most sensitive stage to Cry1Ab toxin, although we have never stated this in any written communication. In fact, 7 years later, we published that the second instar is the most sensitive to Dipel [17].

The young larvae of *N. c-album* feed (peel) alone on the back (dorsal) part of *Urtica* leaves, whereas maize pollen settles down on the front (ventral) surface. Thus, the pollen contact in this case is also low. In contrast, the young larvae of *N. io* and *A. urticae* feed on the front of the leaves. Moreover, *N. io* larvae feed in groups in their first to third instars. They need stimuli in their younger ages for a normal feeding behavior. This peculiar feeding pattern may lead to increased mortality when the larvae sense being exposed to too much maize pollen and begin looking for other places for feeding. The mortality rate of *N. io* larvae is higher when they remain alone [41]. These are reasons why species show such widely different sensitivity to Cry1 toxin. *N. c-album* is nearly sixfold less sensitive to Dipel than *N. io* [17]. None of the current risk analyses [19,20,21,22] may suitably handle differing species sensitivities; instead, they try to build a model for an “imaginable uniform species” when facts are distinctive. There are overly numerous advisers of the concept of “knowledge-based modeling”, and too few active researchers involved in experimental work focus on lepidopteran larval development and behavior in the laboratory, obtaining factual and applicable laboratory and field data [17,18,38,39,40,41].

### 5.2. Toxicity Types of Cry1Ab on Nymphalis io Larvae

**Figure 4:**
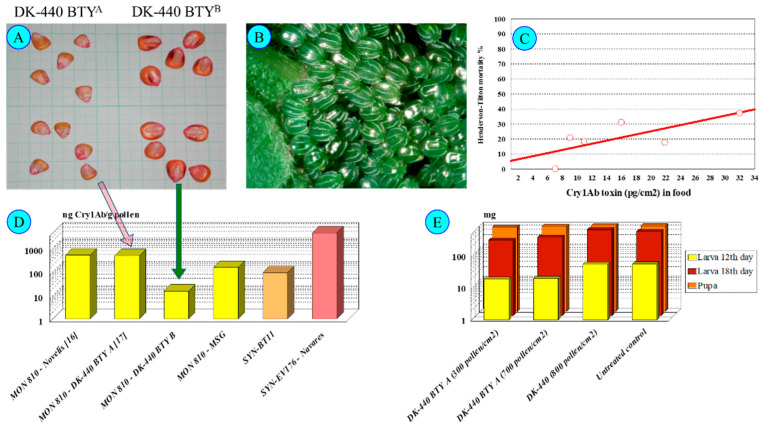
*Inachis io* and DK-440 BTY^A^. Note: (**A**)—DK-440 BTY^A^ and DK-440 BTY^B^ seeds; (**B**)—*Nymphalis io* eggs; (**C**)—*Nymphalis io* larval mortality until pupation treated from L1 on DK-440 BTY^A^ pollen; (**D**)—Cry1Ab toxin-containing cultivars; (**E**)—*Nymphalis io* larval and pupal weight after feeding on *Urtica dioica* leaves containing DK-440 BTY^A^ pollen [5,9,10,11,12,13,16,17,18,30,31].

Pollen with low toxin concentrations (<20 ng Cry1Ab/g pollen) did not exert any effects (in our case, the DK-440 BTY^B^—Figure 4) on *N. io* larvae. In contrast, pollen of higher toxin content (>100–400 ng Cry1Ab toxin/g pollen—DK-440 BTY^A^—Figure 4) exerted substantial observable effects on *N. io* larvae feeding on nettle leaves covered with 300–600 pollen grains/cm^2^ [18]. Low mortality at early larval stages (usually not more than 40% during larval development—Figure 4), delayed early larval development (L1–L3) and lower larval weights until L3 were observed. Moreover, viral infection (cypovirus 2) of the last (L5) instars was unusually frequent. *N. io* larvae appeared to be more sensitive to Cry toxins (Dipel clearly show this sensitivity [17]) than the other species tested (*N. c-album*, *V. atalanta*). A concentration dependence of larval mortality on pollen density is hard to establish, because larvae can actively avoid feeding on leaf parts contaminated with a high density of maize pollen.

A vast number of field studies have been published based on simple pollen densities on weed leaves without Cry1Ab toxin quantification. We do not cite these articles, because the real extent of exposure to Cry1Ab toxin is questionable in these cases.

### 5.3. Variable Larval Sensitivities of Nymphalis io Subpopulations—Cry1 Toxin Resistance

The emergence of resistance to Cry1Ab toxin in insect subpopulations is well known today [18,42,43,44,45,46,47], although mostly is mentioned in relation to pest species only; however, resistance can be developed in non-target insect species as well. In our laboratory, when working with shared egg batches of *N. io* which originated from a distinct pair, using the same simple method as presented earlier [11,18,32], we found that progenies of the same two parents were tolerant to the Cry1Ab toxin content in pollen in six cases, with no mortality found in these cases at up to nearly 600 pollen grains/cm^2^ (MSG cultivars—150 ng Cry1Ab toxin/g pollen). Cry1Ab-resistant *N. io* subpopulations—no surprise—are originally parts of the Hungarian lepidopteran larval population [9,16,18].

## 6. Consequences for a Credible Hazard Assessment

The debate on the effect of Cry1 toxin, which settles on the leaves of *Urtica* species, changes the quality of host plant and causes destruction of the sensitive larval population part of nymphalid species, and is now flaring up again ([19] vs. [20,21,22,45,46]). The fact of the hazard to *N. io* based on laboratory tests cannot be denied [2,5,16,30,40,43], but the extent of the risk is small for *A. urticae* [38,40], *N. c-album* [17] and *V. atalanta* [17]. Nonetheless, the risk is also limited for *N. io*, given the small populations that develop on the edges of cornfields. The published mathematical models are oversimplified and contradict with each other, i.e., are the basis for the corresponding EFSA opinion [19] and others [20,21,22], and do not consider countless relevant biological facts (see Figure 1) clearly established under laboratory tests. Furthermore, the role of the highly variable Cry1Ab toxin contents of MON 810 pollen is completely ignored in this ongoing debate [19,20,21,22,48,49]. Ultimately, assessment is simplified to pollen counting without sufficient environmental analysis [50,51,52,53,54], whereas the relationship to pollen count (even though the Cry1Ab toxin content of pollen stocks are usually different) to larval mortality is not even linear. As seen from our laboratory feeding experiments, *I. io* L1–L3 rejects high maize pollen density and does not consume the area of the leaf vessels where deposition occurs in higher quantities. In our opinion, the likelihood of the actual occurrence of the hazard is low; therefore, the hazard caused by Cry1Ab toxin in maize pollen is in the range of that of any type of *Bacillus thuringiensis* formulation, such as Dipel [55], and certainly does not exceed the hazard of neurotoxic insecticides applied in practice again the corn borer.

As seen from the above discussion, MON 810 cultivars producing more than 100 ng Cry1Ab toxin/g pollen exert an existing hazard to nymphalid larvae. In a valid risk assessment, the question is the likelihood of exposure. In the case of protected species, however, no alteration of the habitat is allowed legally; therefore, the existent hazard is a sufficient cause for restrictions, although in regular cases, risk as a produce of hazard and exposure needs to be considered.

The issue of management of the hazard to wildlife is presently based on environmental law in Europe. This is different from country to country. In Hungary, where *N. io* has a legally protected status, the observed hazard is a sufficient cause to prohibit the cultivation of MON 810 cultivars that produce more than 100 ng Cry1Ab toxin/g pollen. In the case of butterflies not falling under legal environmental protection, the corresponding risk caused by Cry1Ab toxin-containing maize pollen would probably not justify a cultivation moratorium for MON 810. Moreover, regular insecticidal control against *O. nubilalis* and *H. amigera* (these are rare insect pests in Hungary [14]) or *Diabrotica virgifera* (occurring sporadically in Hungary) may cause a similar or higher damage in the protected butterfly populations as the pollen of MON 810. During 2020, abamectin, acetamiprid, beta cyfluthrin*, chlorantraniliprole, chlorpyrifos*, cypermethrin, esfenvalerate, indoxacarb, lambda cyhalothrin, methofenozide and thiacloprid* (note: * indicates the active ingredient has not been applied in formulated pesticide products in 2021) were listed as authorized active ingredients for insect control in maize fields in Hungary. This is a very sharp contradiction—between Cry1 toxin-containing pollen and authorized insecticide treatments—in cases of protected nymphalid species in Hungary.

The interest in maize pollen dispersal has originated in different areas: (*i*) gene flow resulting in intraspecific hybrids in European seed production [9,23,56,57]; (*ii*) direct environmental impacts on sensitive non-target species as far as some meters from maize fields in the case of varieties producing Cry toxins; and (*iii*) the effects on honeybees collecting maize pollen as a protein source to feed their larvae and thereby contaminating honey.

In the case of intraspecific hybridization, shorter or longer isolation zones are involved. Nonetheless, despite the short pollen lifetime and low pollen numbers in the air, the GM-pollen source can result in intraspecific hybridization with maize hybrids and interspecific hybridization with wild teosinte relatives in Central America. Most of the related publications consider the critical hybridization zone being several hundred meters long. In our experiments, we found ~10% colored seeds at 200 m in the case of a white maize cultivar. We did not find—using genetically dominant blue colored maize as a pollen source—intraspecific hybridization near 800 m in a valley (Nagykovácsi—Budapest, Ady-liget) and in the direction of prevalent wind [57].

## Data Availability

Not applicable.
